# Supplementary material for: DNA methylation as a contributor to dysregulation of STX6 and other frontotemporal Lobar degeneration genetic risk-associated loci
Source: Acta Neuropathol Commun. 2025 Jul 5;13:148. doi: 10.1186/s40478-025-02071-3 (PMC12228266; doi:10.1186/s40478-025-02071-3)
Supplement: Supplementary file 2 — Supplementary Material 2 [file 40478_2025_2071_MOESM2_ESM.docx]

**DNA methylation as a contributor to dysregulation of *STX6* and other frontotemporal lobar degeneration genetic risk-associated loci**

Naiomi Rambarack^1^, Katherine Fodder**^1^**, Megha Murthy^2^**, Christina Toomey^2,3^, Rohan de Silva^2,4^, Peter Heutink^5^, Jack Humphrey^6^, Towfique Raj^6^, Tammaryn Lashley^1^, Conceição Bettencourt^1*^**

1. Department of Neurodegenerative Disease, UCL Queen Square Institute of Neurology, London, UK
2. Department of Clinical and Movement Neurosciences, UCL Queen Square Institute of Neurology, London, UK
3. The Francis Crick Institute, London, UK
4. Reta Lila Weston Institute, UCL Queen Square Institute of Neurology, London, UK
5. German Center for Neurodegenerative Diseases, Tübingen, Germany
6. Nash Family Department of Neuroscience and Friedman Brain Institute, Icahn School of Medicine at Mount Sinai, New York, NY USA

*Corresponding Author:
Conceição Bettencourt, PhD
Department of Neurodegenerative Disease
UCL Queen Square Institute of Neurology

1 Wakefield Street
London WC1N 1PJ
United Kingdom

Email [c.bettencourt@ucl.ac.uk](mailto:c.bettencourt@ucl.ac.uk)

**Supplementary Figures**


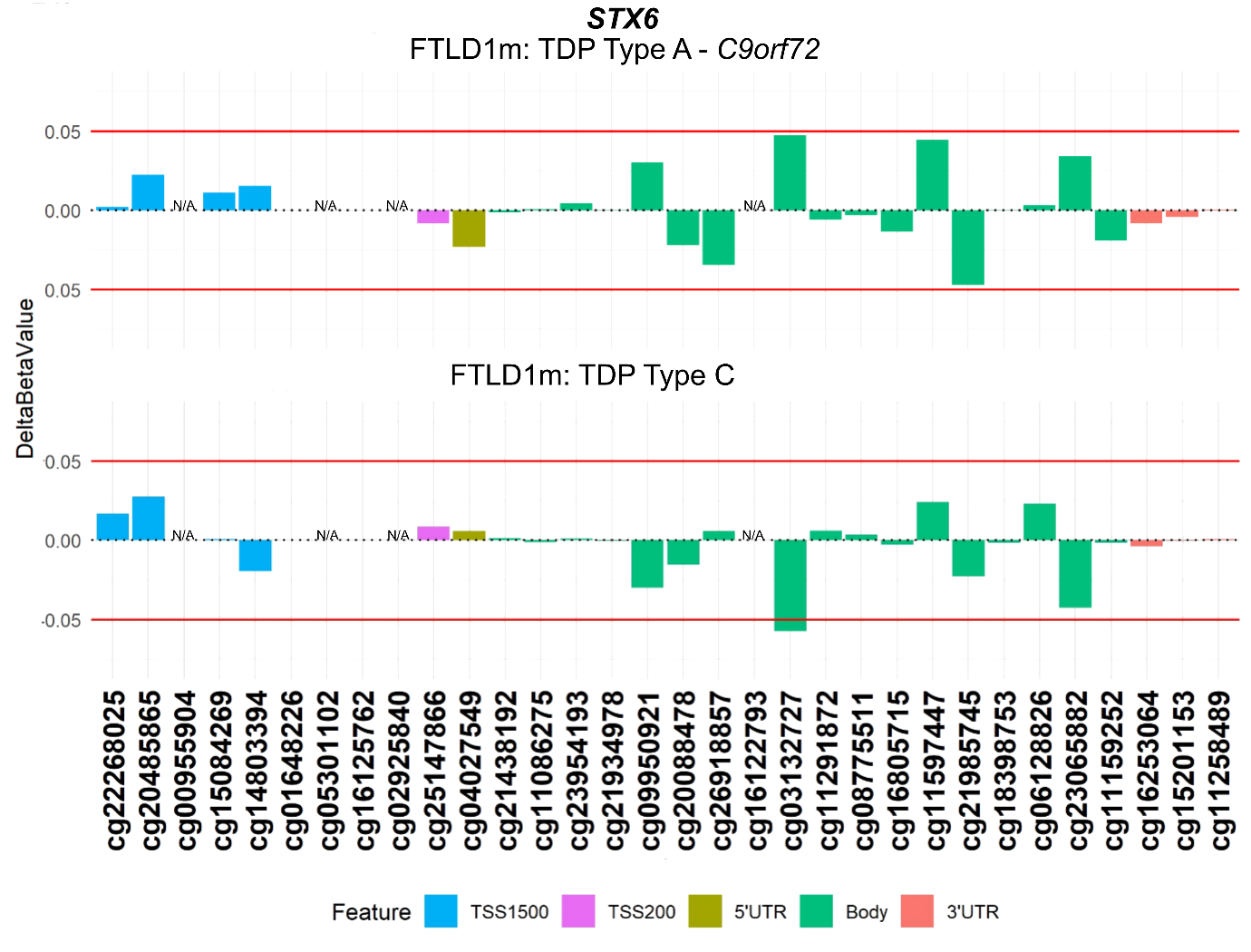
 **Supplementary Fig. 1** **Analysis of DNA methylation patterns across the *STX6* locus in FTLD1m**. Note: CpGs at the promoter region showing changes in other FTLD subtypes (cg02925840 and cg05301102) were not present in this dataset due to their exclusion during data quality control pre-processing. FTLD1m – frontotemporal lobar degeneration DNA methylation cohort 1, TSS – transcription start site; TSS200 – 0–200 bases upstream of TSS; TSS1500 – 200-1500 bases upstream of TSS; UTR – untranslated region. NA – These CpGs were not available in the specified dataset due to differences in the methylation array (450K or EPIC) or removal during quality control.


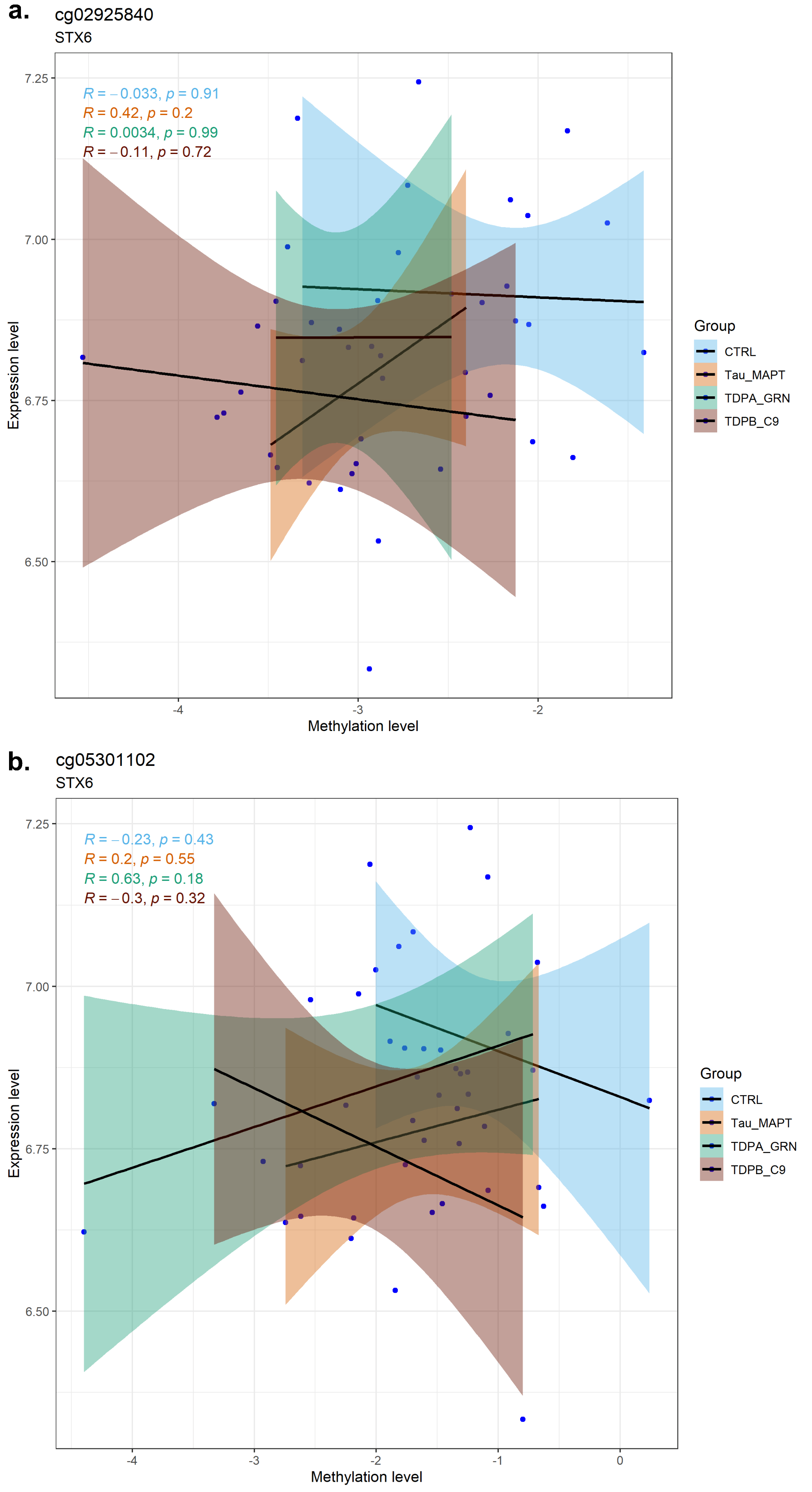


**Supplementary Fig. 2** **DNA methylation-gene expression correlations in FTLD2 datasets for the top two CpGs mapping to *STX6* promoter**. **(a)** cg02925840 and **(b)** cg05301102. Log2-transformed gene expression data is shown in the *y*-axis, and DNA methylation levels (M-values) are shown in the *x*-axis. FTLD2 – frontotemporal lobar degeneration cohort 2.


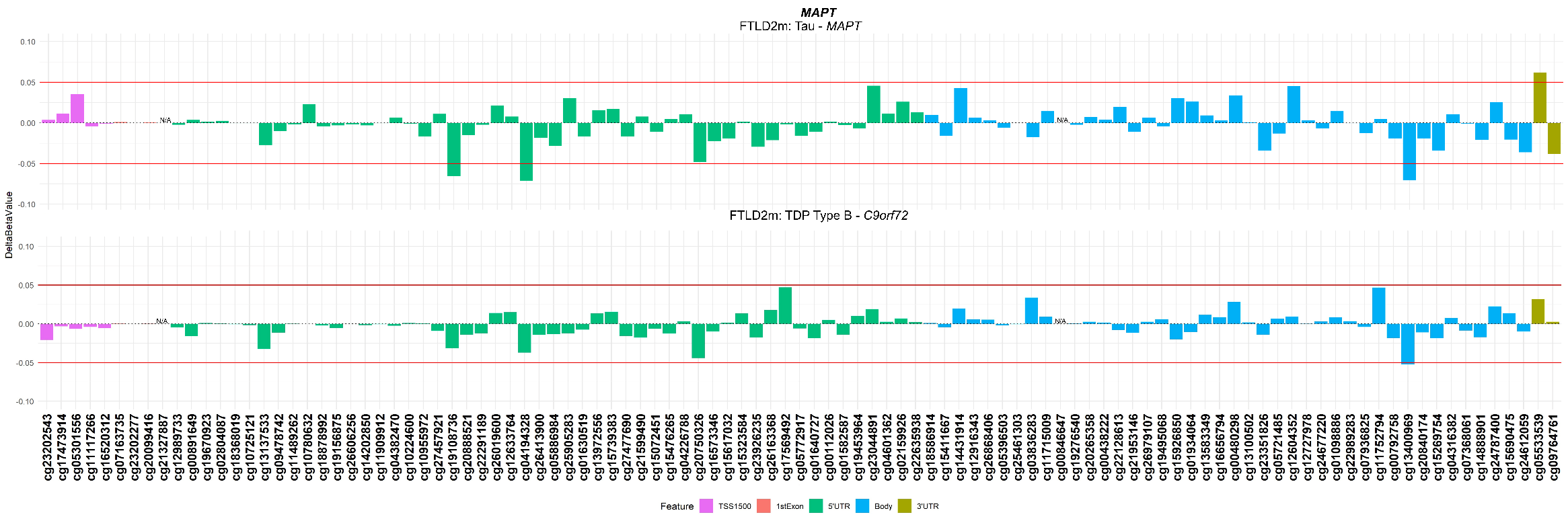


**Supplementary Fig. 3 Analysis of DNA methylation patterns across the *MAPT* locus in *GRN* and *C9orf72* cases from the FTLD2m cohort**. Note: No probes met the significance threshold (p<0.05) in the FTLD-TDP Type A *GRN* or Type B *C9orf72* cases compared to controls. FTLD2m – frontotemporal lobar degeneration DNA methylation cohort 2, TSS – transcription start site; TSS200 – 0–200 bases upstream of TSS; TSS1500 – 200-1500 bases upstream of TSS; UTR – untranslated region. NA – These CpGs were not available in the specified dataset due to differences in the methylation array (450K or EPIC) or removal during quality control.


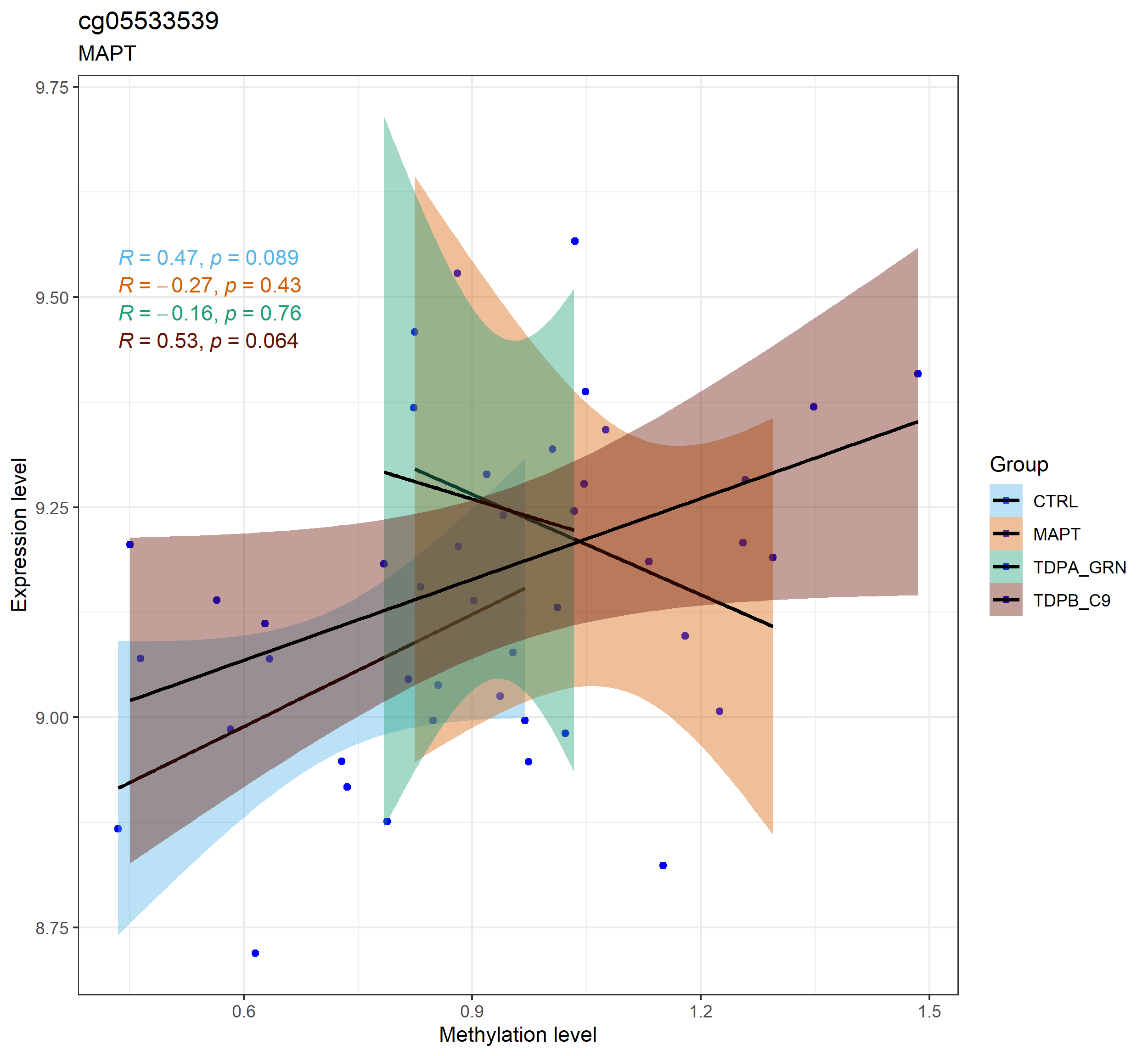


**Supplementary Fig. 4** **DNA methylation-gene expression correlations in the FTLD2 datasets for the *MAPT* 3’UTR CpG differentially methylated in *MAPT* mutation carriers (cg05533539)**. Log2-transformed gene expression data is shown in the *y*-axis, and DNA methylation levels (M-values) are shown in the *x*-axis. FTLD2 – frontotemporal lobar degeneration cohort 2; 3’UTR – 3’ untranslated region.


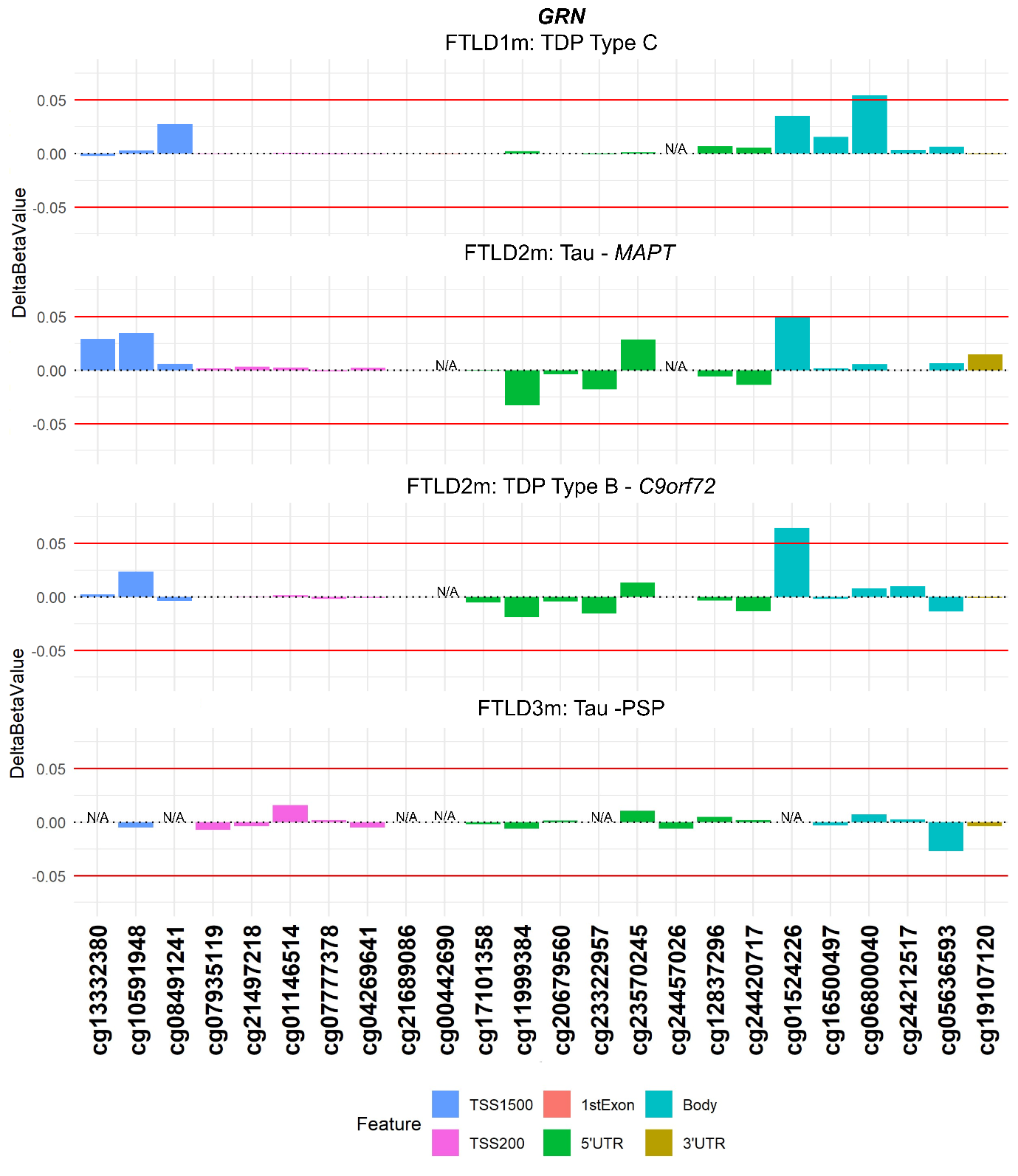


**Supplementary Fig. 5 Analysis of DNA methylation patterns across the *GRN* locus in non-TDP Type A cases**. Note: No probes showed dysregulated methylation (absolute delta-beta ≥5%, p<0.05) at the promoter region in other FTLD-TDP types or FTLD-Tau cases. TSS – transcription start site; TSS200 – 0–200 bases upstream of TSS; TSS1500 – 200-1500 bases upstream of TSS; UTR – untranslated region. NA – These CpGs were not available in the specified dataset due to differences in the methylation array (450K or EPIC) or removal during quality control.


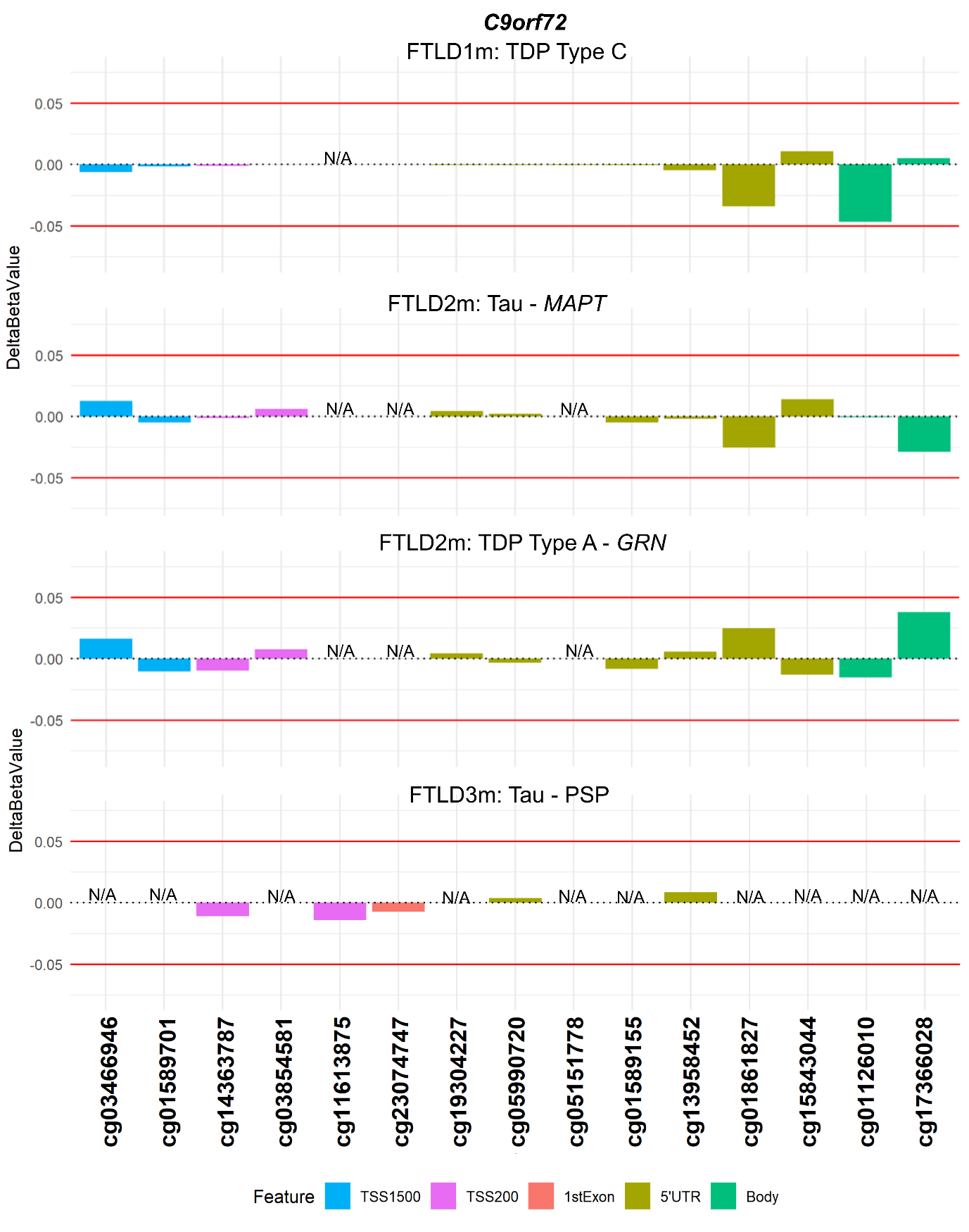


**Supplementary Fig. 6 Analysis of DNA methylation patterns across the *C9orf72* locus in non-mutation carriers**. Note: No probes at any region showed an absolute delta-beta, i.e. mean difference when compared to controls, of ≥5%. TSS – transcription start site; TSS200 – 0–200 bases upstream of TSS; TSS1500 – 200-1500 bases upstream of TSS; UTR – untranslated region. NA – These CpGs were not available in the specified dataset due to differences in the methylation array (450K or EPIC) or removal during quality control.
